# Preserved immune functionality and high CMV-specific T-cell responses in HIV-infected individuals with poor CD4^+^ T-cell immune recovery

**DOI:** 10.1038/s41598-017-12013-2

**Published:** 2017-09-15

**Authors:** Elisabet Gómez-Mora, Elisabet García, Victor Urrea, Marta Massanella, Jordi Puig, Eugenia Negredo, Bonaventura Clotet, Julià Blanco, Cecilia Cabrera

**Affiliations:** 1 IrsiCaixa AIDS Research Institute, Institut de Recerca Germans Trias i Pujol (IGTP), Hospital Universitari Germans Trias i Pujol, Universitat Autonoma de Barcelona, 08916 Badalona, Barcelona Spain; 2 Université de Montréal, Faculté de Médecine, Department of microbiology, infectiology and immunology, Centre de Recherche du CHUM, Montréal, QC Canada; 3 Fundació Lluita contra la SIDA, Hospital Universitari Germans Trias i Pujol, 08916 Badalona, Barcelona Spain; 4 Universitat de Vic-UCC, 08500 Vic, Barcelona Spain

## Abstract

Poor CD4^+^ T-cell recovery after cART has been associated with skewed T-cell maturation, inflammation and immunosenescence; however, T-cell functionality in those individuals has not been fully characterized. In the present study, we assessed T-cell function by assessing cytokine production after polyclonal, CMV and HIV stimulations of T-cells from ART-suppressed HIV-infected individuals with CD4^+^ T-cell counts >350 cells/μL (immunoconcordants) or <350 cells/μL (immunodiscordants). A group of HIV-uninfected individuals were also included as controls. Since CMV co-infection significantly affected T-cell maturation and polyfunctionality, only CMV^+^ individuals were analyzed. Despite their reduced and skewed CD4^+^ T-cell compartment, immunodiscordant individuals showed preserved polyclonal and HIV-specific responses. However, CMV response in immunodiscordant participants was significantly different from immunoconcordant or HIV-seronegative individuals. In immunodiscordant subjects, the magnitude of IFN-γ^+^ CD8^+^ and IL-2^+^ CD4^+^ T-cells in response to CMV was higher and differently associated with the CD4^+^ T-cell maturation profile., showing an increased frequency of naïve, central memory and EMRA CMV-specific CD4^+^ T-cells. In conclusion, CD4^+^ and CD8^+^ T-cell polyfunctionality was not reduced in immunodiscordant individuals, although heightened CMV-specific immune responses, likely related to subclinical CMV reactivations, may be contributing to the skewed T-cell maturation and the higher risk of clinical progression observed in those individuals.

## Introduction

Combination antiretroviral therapy (cART) with effective control of viral replication and subsequent immunologic reconstitution has dramatically improved the health of HIV-infected individuals, resulting in a reduction in HIV-related morbidity and mortality^1^. However, despite persistent virus suppression, about 15–30% of treated HIV-infected individuals fail to achieve optimal CD4^+^ T-cell reconstitution, referred as immunological non-responders or immunodiscordant individuals^2,3^. Several factors have been associated with a poor CD4^+^ T-cell immune recovery (reviewed in ref.^4^), among others altered thymic production^5,6^, low nadir CD4 counts^7^, older age^8^, high levels of immune activation^5,7,9^ and increased cell death^5,7^. Additionally, immunodiscordant individuals show a skewed T-cell maturation profile^10–13^, increased expression of markers of replicative senescence (CD28^+^CD57^+^)^6,13,14^ and high frequencies of programmed cell death protein-1 (PD-1)-expressing CD4^+^ T-cells^5,15^, a phenotype associated with immune exhaustion, and defined by loss of effector functions and proliferative capacity. However, it is unclear how these changes affect the functional diversity (i.e. polyfunctionality) of CD4^+^ and CD8^+^ T-cells in immunodiscordant individuals.

Cytomegalovirus (CMV) infection in healthy individuals is usually asymptomatic and results in latent infection. CMV co-infection is highly prevalent in the HIV-infected population (between 75 and 100%)^16^ and episodes of CMV-reactivation are increased, affecting morbidity and mortality^17^. CMV infection is also associated with significant changes in the composition of the T-cell repertoire, accelerated T-cell immunosenescence and immune exhaustion^18,19^. In particular, CMV has been described as a major contributor to the increased immune activation and senescence in HIV^+^ individuals with poor CD4^+^ T-cell recovery^20–22^. Furthermore, increased CMV-specific antibodies and/or T-cells have been associated with atherosclerosis and impaired CD4^+^ T-cell reconstitution and progression in HIV-infected individuals on treatment^23–27^. However, CMV-specific T-cell responses in individuals with poor CD4^+^ T-cell recovery have not been completely characterized.

We hypothesized that skewed CD4^+^ T-cell maturation and increased exhaustion could be factors contributing to an impaired T-cell polyfunctionality in immunodiscordant individuals. Therefore, in the present study we analyzed cellular immune response of CMV-seropositive HIV-infected individuals with different CD4^+^ T-cell recovery upon virologically suppressive cART. The frequency, functional capacity and differentiation profile of CD4^+^ and CD8^+^ T-cells after PMA and ionomycin, CMV and HIV stimulation was evaluated.

## Results

### Participant characteristics

A total of 43 HIV-infected individuals were included: 25 participants were classified as immunoconcordants and 18 as immunodiscordants (Table 1). Both HIV-infected groups were similar in age, gender, prevalence of HCV, time since diagnosis and treatment conditions (Table 1). As per inclusion criteria, significantly lower absolute CD4^+^ T-cell counts were observed in the immunodiscordant group than in the immunoconcordant group. In addition, also lower nadir CD4^+^ T-cell counts and CD8^+^ T-cell counts were observed in the immunodiscordant group. Although not significant, a higher proportion of CMV-seropositive (CMV^+^) individuals were found in the HIV-infected group than in the HIV-uninfected control group. None of the participants had detectable CMV viral load in urine samples as assessed using quantitative CMV-PCR.

**Table 1:** Main clinical and immunological characteristics of participants.

	HIV^+^ Subgroups					
	Uninfected controls (n = 21)	HIV^+^-Infected ART-Treated (n = 43)	*p*-value^1^	Immuno- concordant (n = 25)	Immuno- discordant (n = 18)	*p-*value^2^
Gender (male); n (%)	11 (50)	38 (88)	***0***.***0034***	21 (84)	17 (94)	*0*.*38*
Age (years); Median [IQR]	41 [38-–52]	50 [48–57]	***0***.***0011***	49 [48–54]	50 [48–54]	*0*.*63*
CMV-seropositive individuals; n (%)	13 (62)	36 (84)	*0*.*13*	21 (84)	15 (83)	*1*
Absolute CD4^+^ T-cell count (cells/µL); Median [IQR]	754 [545–994]	441 [248–909]	***0***.***037***	849 [658–1214]	254 [166–306]	**<** ***0***.***0001***
Absolute CD8^+^ T-cell count (cells/µL); Median [IQR]	345 [306–376]	614 [405–841]	**<** ***0***.***0001***	733 [572–1151]	398 [298–658]	***0***.***0014***
CD4^+^ Nadir (cells/µL); Median [IQR]		122 [51–191]		167 [74–282]	61 [15–134]	***0***.***0014***
Time since HIV diagnosis (years); Median [IQR]		17 [11–22]		21 [17–27]	24 [17–28]	*0*.*96*
Time on ART (years); Median [IQR]		14 [9–17]		17 [15–22]	17 [13–20]	*0*.*37*
HCV co-infection; n (%)		17 (39)		10 (40)	7 (39)	*1*

^1^Comparison of HIV-infected ART-treated and uninfected individuals (Mann-Withney U or Fisher exact test). ^2^Comparison of immunoconcordant and immunodiscordant HIV-infected ART-treated individuals (Mann-Withney U or Fisher exact test).

### Impact of CMV infection on T-cell maturation and polyfunctionality

**Figure 1:**
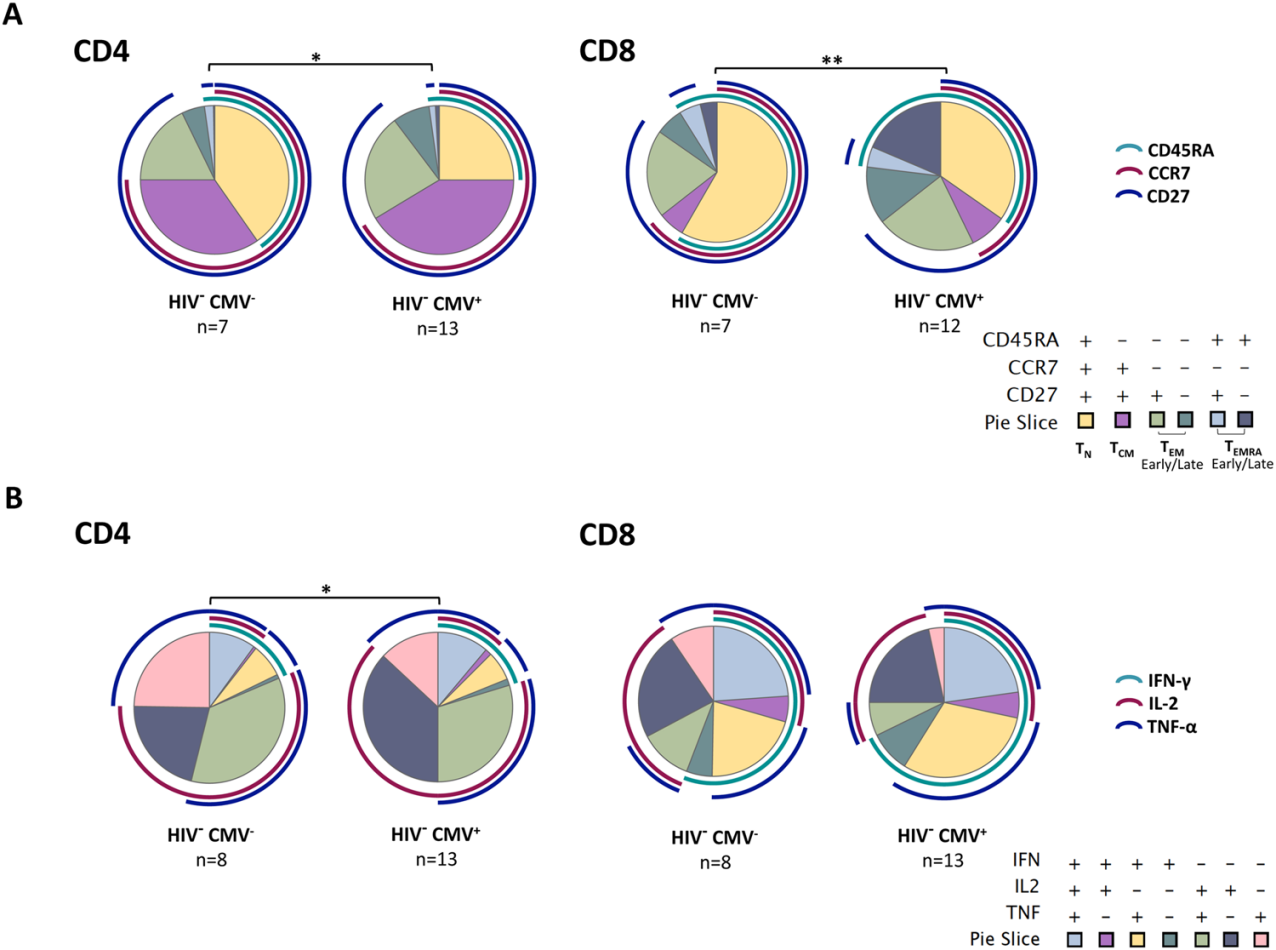
Phenotypic and functional changes induced by CMV infection. (**A**) CD4^+^ and CD8^+^ T-cell differentiation profile in HIV-uninfected CMV-seropositive and -seronegative individuals. The differential expression of CD45RA, CCR7 and CD27 by CD4^+^ and CD8^+^ T-cells was analyzed by boolean gating. Pie charts illustrate relative proportions of each of the different subsets in CMV-seropositive and CMV-seronegative. (**B**) CD4^+^ and CD8^+^ T-cell responses to PMA/ionomycin stimulation in HIV-uninfected individuals stratified by CMV serostatus. Polyfunctional profiles of CD4^+^ and CD8^+^ T-cells responding to PMA/ionomycin are showed. Detail of the 3-function combinations (IFN-γ, IL-2 and TNF-α) of CD4^+^ and CD8^+^ T-cells responding to PMA/ionomycin is depicted on the bottom of the figure. Statistical comparisons of the profiles were performed by partial permutation tests, using Spice software (*p < 0.05, **p < 0.01). HIV^−^: HIV-uninfected individuals; CMV^−^: CMV-seronegative individuals; CMV^+^: CMV-seropositive individuals.

We observed a significant impact of CMV serostatus in the phenotypic distribution of CD4^+^ and CD8^+^ T-cells (p = 0.04 and p = 0.01, respectively, Fig. 1A and gating strategy in Figure S1). CMV-seropositive HIV-uninfected individuals showed decreased naïve cells and increased CD27^−^ subsets (T_EM_ and T_EMRA_ cells) than CMV-seronegative. Although a trend was seen, there was no significant difference in the age between CMV^−^ and CMV^+^ populations (median (IQR): 38 (36–45) and 46 (41–54), respectively p = 0.07). Moreover, significant differences in the polyfunctional profile after PMA/Ionomycin stimulation of CD4^+^ T-cells (p = 0.049) was also observed between CMV-seropositive and CMV-seronegative HIV-uninfected individuals (Fig. 1B). Phenotypic and functional changes by CMV infection in the HIV^+^ ART-treated population could not be analyzed because the low number of HIV^+^CMV^−^ individuals recruited. However, to avoid the bias induced by the potential immunological impact of CMV infection, only CMV-seropositive individuals were included for the rest of the analyses. Table 2 summarizes the main characteristics of CMV-seropositive individuals. Unlike the whole cohort (Table 1) no differences in age or CD4^+^ T-cell counts were observed between HIV-uninfected and HIV-infected CMV-seropositive groups. No other differences were observed between CMV-seropositive populations (Table 2) in comparison with the whole cohort (Table 1).

**Table 2:** Characteristics of CMV^+^ participants.

	HIV^+^ Subgroups					
	CMV^+^ Uninfected controls (n = 13)	CMV^+^ HIV^+^-Infected ART-Treated (n = 36)	*p*-value^1^	CMV^+^ Immuno- concordant (n = 21)	CMV^+^ Immuno- discordant (n = 15)	*p-*value^2^
Gender (male); n (%)	7 (54)	30 (86)	***0***.***048***	17 (81)	14 (93.3)	*0*.*38*
Age (years); Median [IQR]	46 [41–54]	49 [48–54]	*0*.*078*	49 [48–54]	50 [48–54]	*0*.*63*
Absolute CD4^+^ T-cell count (cells/µL); Median [IQR]	643 [542–823]	451 [257–963]	*0*.*38*	888 [661–1225]	251 [166–351]	<***0***.***0001***
Absolute CD8^+^ T-cell count (cells/µL); Median [IQR]	344 [298–366]	668 [409–906]	<***0***.***0001***	753 [618–1333]	398 [333–654]	***0***.***0014***
CD4^+^ Nadir (cells/µL); Median [IQR]		116 [51–188]		163 [65–282]	61 [20–134]	***0***.***01***
Time since HIV diagnosis (years); Median [IQR]		22 [18–29]		22 [18–28]	26 [19–22]	*0*.*46*
Time on ART (years); Median [IQR]		18 [15–24]		17 [15–20]	20 [12–24]	*0*.*9*
HCV co-infection; n (%)		14 [39]		8 (38)	6 (40)	*1*

^1^Comparison of CMV^+^HIV-infected ART-treated and CMV^+^ uninfected individuals (Mann-Withney U or Fisher exact test). ^2^Comparison of CMV^+^ immunoconcordant and CMV^+^ immunodiscordant HIV-infected ART-treated individuals (Mann-Withney U or Fisher exact test).

### Polyfunctional profile of CD4^+^ and CD8^+^ T-cell responses according to CD4^+^ T-cell recovery

**Figure 2:**
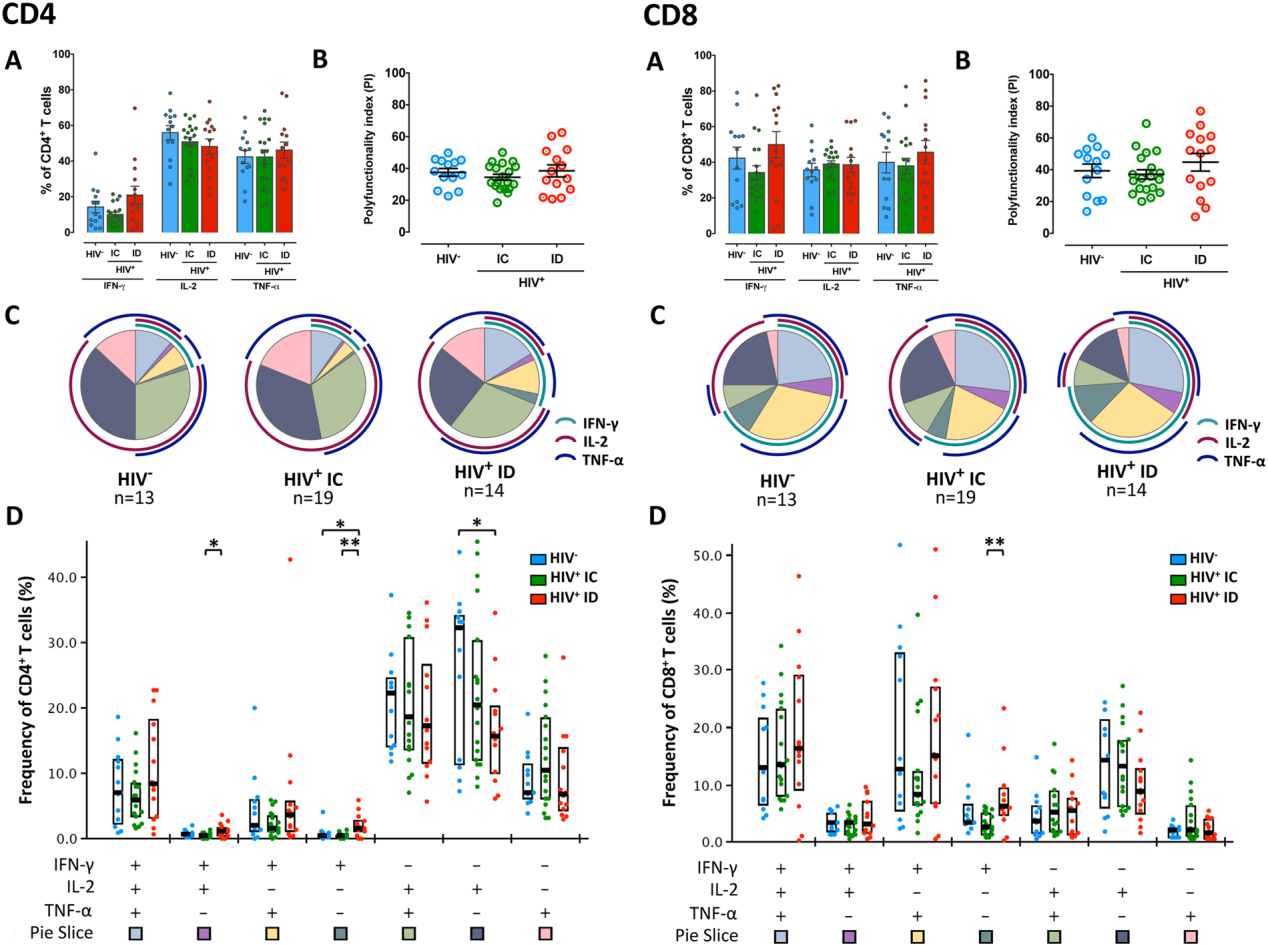
CD4^+^ and CD8^+^ T-cell responses to PMA/ionomycin stimulation. (**A**) The frequency of CD4^+^ and CD8^+^ T-cells positive for each cytokine (IFN-γ, IL-2 and TNF-α) is represented (mean and SEM). (**B**) Polifunctionality index (PI) of HIV-uninfected individuals, immunoconcordant and immunodiscordant individuals (Mean and SEM are represented). (**C**) Pie charts representing the polyfunctional profile of CD4^+^ and CD8^+^ T-cells from HIV-uninfected individuals, immunoconcordant and immunodiscordant individuals. Arcs depict cytokine makeup within pie slice. (**D**) Dot graphs indicate percentage of responding cells based on different combinations of cytokine function. The combination of functions studied (pie slice) is indicated in the graph below (Median and IQR are represented). Nonparametric Mann-Whitney U test was used to analyze differences between groups (*p < 0.05, **p < 0.01). HIV^−^: CMV^+^ HIV-uninfected individuals; HIV^+^: CMV^+^ HIV-infected ART-treated individuals; IC: CMV^+^ immunoconcordant individuals; ID: CMV^+^ immunodiscordant individuals.

To evaluate the T-cell functional diversity in the three analyzed groups (HIV-negative, HIV^+^ immunoconcordant and HIV^+^ immunodiscordant), frequencies of CD4^+^ and CD8^+^ T-cells that expressed IFN-γ, IL-2 and TNF-α following the strong non-specific stimulation with PMA/ionomycin were evaluated (Fig. 2). CD4^+^ T-cells from immunodiscordant subjects secreted slightly higher, but not significant total levels of IFN-γ and similar IL-2 and TNF-α levels than HIV-negative and immunoconcordant individuals (Fig. 2A, Panel CD4). Consistently, no significant differences between the three groups were observed when the polyfunctional index (Fig. 2B, Panel CD4) or the SPICE polyfunctional profile (Fig. 2C, Panel CD4) was compared among groups. However, in a more detailed analysis, the immunodiscordant group showed an increase in all the IFN-γ^+^ subsets, reaching statistical significance in the bifunctional subset displaying IFN-γ and IL-2 and the subset producing IFN-γ alone (Fig. 2D, Panel CD4). Conversely, a lower proportion of the monofunctional IL-2^+^ subset was found in the immunodiscordant group compared with HIV-uninfected individuals (Fig. 2D, Panel CD4).

Likewise, immunodiscordant individuals showed a higher, although no significant, proportion of total CD8^+^ T-cells producing IFN-γ in comparison with immunoconcordant individuals (p = 0.05) with no differences in the polyfunctional index or in the SPICE polyfunctional CD8^+^ T-cell profile between groups (Fig. 2, Panel CD8). All these data suggest that, after a global stimulation, the CD4^+^ and CD8^+^ T-cell polyfunctionality profile is preserved in immunodiscordant individuals compared with immunoconcordant and HIV-negative individuals, although a skewed response towards high IFN-γ production could be observed in immunodiscordant individuals.

### Functional T-cell responses mediated by IFN-γ and IL-2 against CMV and HIV

**Figure 3:**
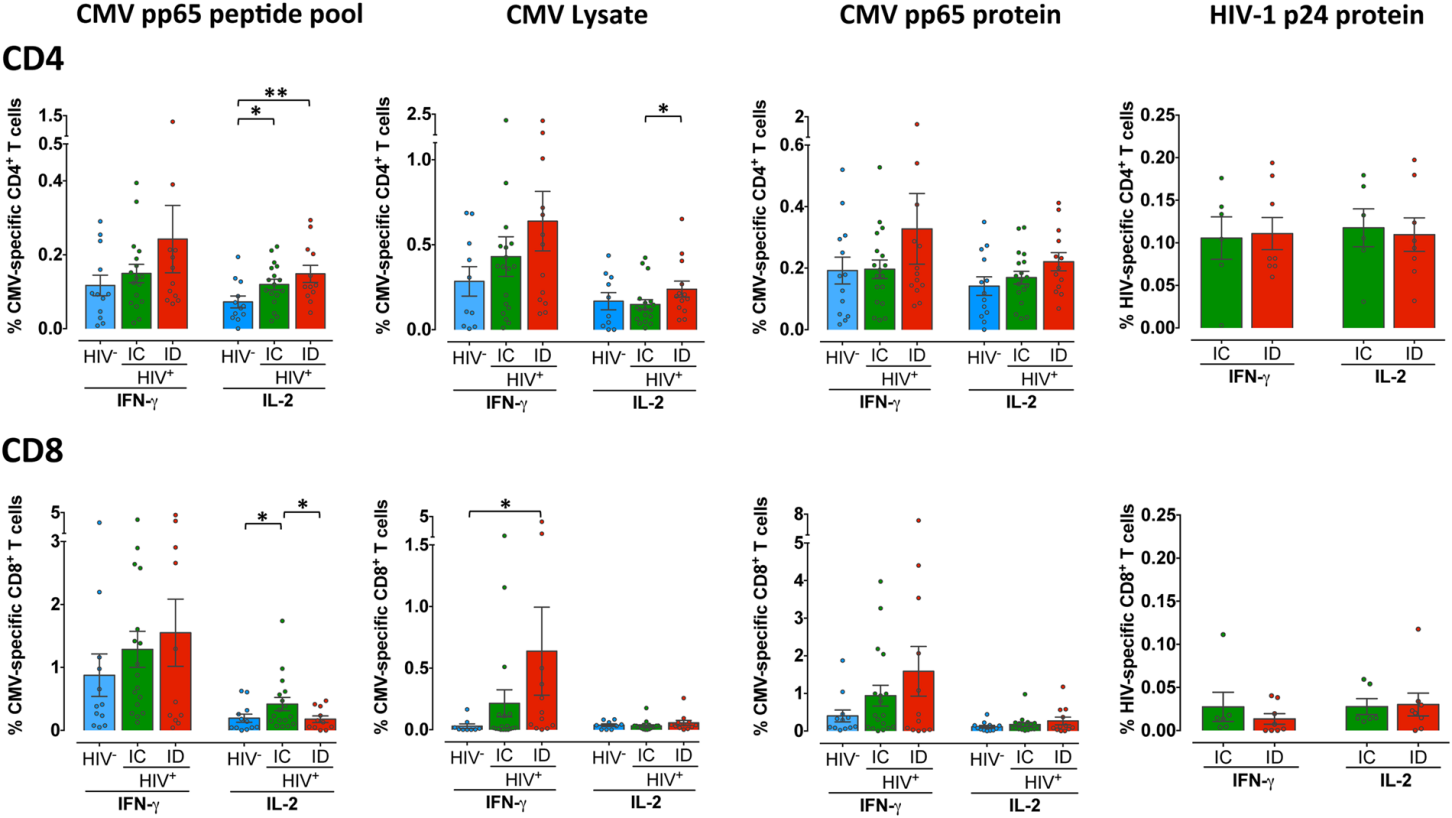
Functional CD4^+^ and CD8^+^ T-cell responses to CMV and HIV antigens. PBMCs were stimulated with different CMV antigenic preparation and the HIV p24 recombinant protein. The specific CD4^+^ and CD8^+^ T-cell responses were measured by flow cytometry in CMV^+^ HIV-uninfected individuals (blue bars, n = 12), CMV^+^ immunoconcordant individuals (green bars, n = 17 and n = 6 for CMV antigens and p24, respectively) and CMV^+^ immunodiscordant individuals (red bars, n = 12 and n = 8 for CMV antigens and p24, respectively). Response magnitudes are reported as the percentages of CD4^+^ and CD8^+^ T-cells producing IFN-γ and IL-2 after background subtraction. Bars indicate mean values ± SEM. Individual data of all subjects are represented by dots. Differences were tested using Mann-Whitney U nonparametric test (*p < 0.05).

To further investigate the functional properties of the different groups of individuals, CMV-specific immune responses (IFN-γ and IL-2 production) were analyzed. Three different CMV antigens were used: a whole CMV viral lysate, containing a high diversity of T-cell epitopes, a pp65 peptide pool, and the pp65 recombinant protein. A similar pattern of total IFN-γ^+^ and IL-2^+^ CMV-specific CD4^+^ and CD8^+^ T-cells was observed in response to all the CMV antigens used, although the magnitude was different between antigens in the two T-cell populations (Fig. 3). As expected, the IFN-γ response was higher in CD8^+^ T-cells than in CD4^+^ T-cells after stimulation with all the stimuli and the proportion of IFN-γ ^+^ cells, in both CD4^+^ and CD8^+^ T-cells, exceeded that of IL-2^+^ cells. Between groups, a tendency to higher total IFN-γ-secreting cells in the immunodiscordant group was observed, although only in CD8^+^ T-cells stimulated with CMV viral lysate reached significant differences in comparison with the HIV-negative group (Fig. 3). The proportion of total IL-2^+^ pp65 peptide pool-specific CD4^+^ T-cells was significantly increased in both immunoconcordant and immunodiscordant individuals in comparison with the HIV-negative group. IL-2^+^ cells were also significantly higher in immunodiscordant than in immunoconcordant individuals after CMV viral lysate stimulation. In CD8^+^ T-cells, an increase of IL-2^+^ cells was observed in immunoconcordant individuals in comparison to both immunodiscordant and the HIV-negative group in presence of CMV pp65 peptide pool. Furthermore, an association between cellular and humoral CMV-specific immune responses was found. Higher CMV IgG levels were observed in immunodiscordant individuals with a significant association between IL-2^+^ CMV-specific CD4^+^ T-cell response and the anti-CMV IgG titers (Spearman r = 0.34; p = 0.03) (data not shown).

Regarding HIV-specific responses, after stimulation with the HIV-1 p24 recombinant protein, the mean frequency of HIV-specific CD4^+^ T-cells was lower than the CMV-responses and no differences were observed between immunoconcordant and immunodiscordant individuals (Fig. 3). The CD8^+^ T-cell response to the HIV p24 recombinant protein appeared to be negligible with a mean frequency of 0.016 of the total CD8^+^ subset responding to the protein. Thus, the results obtained by the analysis of distinct CMV Ag-specific responses indicated that an increased CMV-specific response could be observed in HIV-positive individuals and the response was highest in immunodiscordant individuals.

### Functional analysis of different memory CD4^+^ and CD8^+^ T-cell subsets

**Figure 4:**
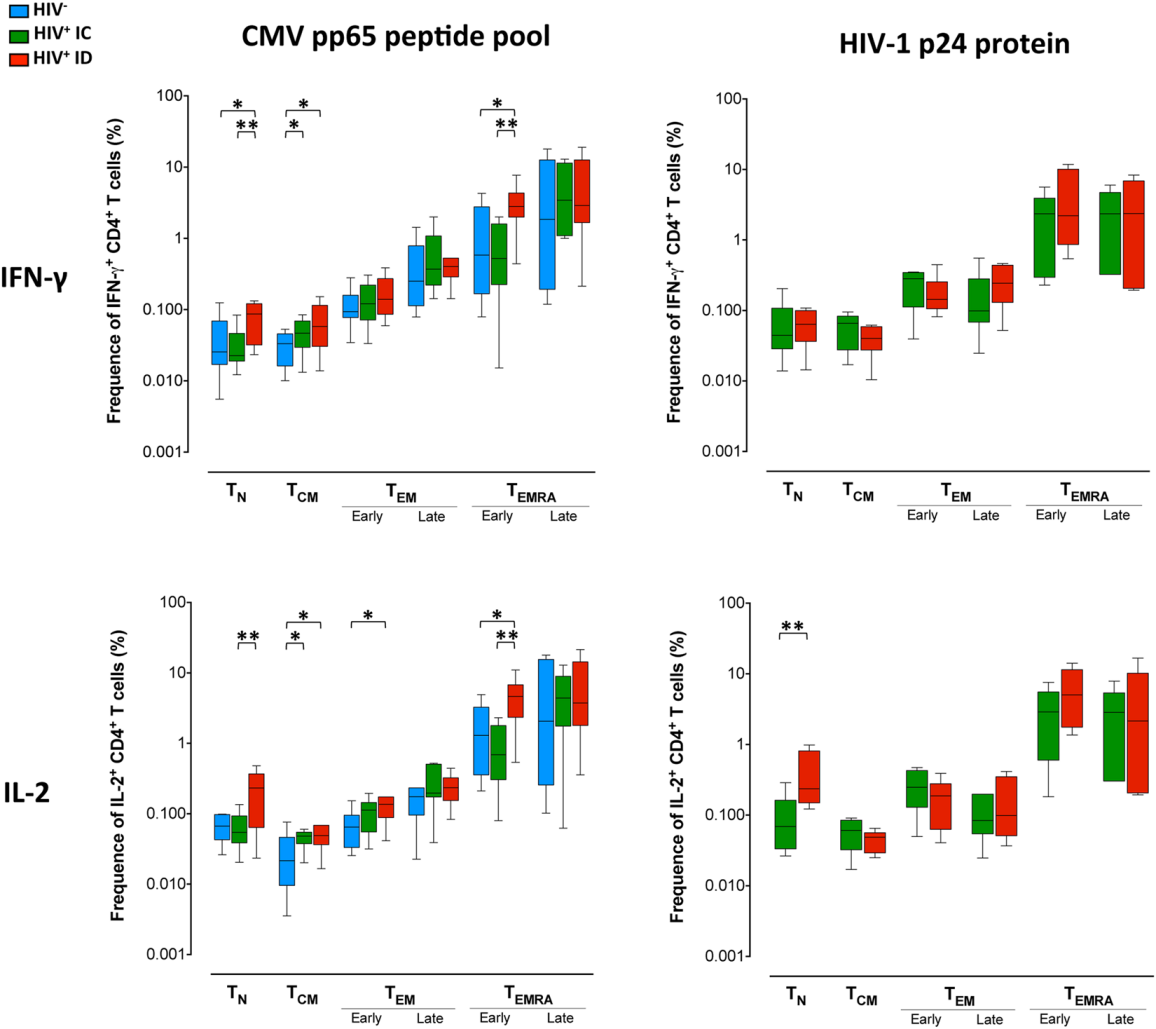
Functionality of CMV and HIV-specific memory CD4^+^ and CD8^+^ T-cell subsets. The differential expression of CD45RA, CCR7 and CD27 by CD4^+^ and CD8^+^ T-cells was analyzed by boolean gating. Based on the expression of these surface markers we were able to discriminate eight different subpopulations expressing each possible combination of markers: naïve (T_N_, CD45RA^+^CCR7^+^CD27^+^), central memory (T_CM_, CD45RA^−^CCR7^+^CD27^+^), early and late effector memory (T_EM early_, CD45RA^−^CCR7^-^CD27^+^ and T_EM late_, CD45RA^−^CCR7^−^CD27^−^), and early and late effector memory re-expressing CD45RA T-cells (T_EMRA early_, CD45RA^+^CCR7^−^CD27^+^ and T_EMRA late_, CD45RA^+^CCR7^−^CD27^−^). Other intermediate phenotypes (CD45RA^+^CCR7^+^CD27^−^ and CD45RA^−^CCR7^+^CD27^−^), which can not as yet be ascribed to a specific subpopulation or to a functionally unique subset, observed in low percentages are not shown. The frequency of IFN-γ^+^ and IL-2^+^ CMV and HIV-specific T-cells across distinct subsets are shown. CMV^+^ HIV-uninfected individuals (blue bars, n = 12), CMV^+^ immunoconcordant individuals (green bars, n = 16 and n = 6 for pp65 peptide pool and p24, respectively) and CMV^+^ immunodiscordant individuals (red bars, n = 11 and n = 8 for pp65 peptide pool and p24, respectively). The median and tukey ranges are shown for each group. Differences were tested using Mann-Whitney U nonparametric test (*p < 0.05, **p < 0.01).

In our study, the overall profile of CD4^+^ and CD8^+^ T-cell maturation was significantly different between immunodiscordant and immunoconcordant individuals, with a significant decrease in the percentage of both naïve CD4^+^ and CD8^+^ T-cells and an increase in different memory subsets (Figure S2). To determine whether this disturbed maturational profile is also accompanied by changes in functionality, we next evaluated the capacity to produce IFN-γ and IL-2 after CMV or HIV p24 stimulation of different memory CD4^+^ and CD8^+^ T-cell subsets. Remarkably all memory CD4^+^ T-cell subsets of immunodiscordant individuals were functional, able to produce IFN-γ and IL-2 after CMV stimulation. Moreover, increased proportions of several memory CD4^+^ T-cell subsets IFN-γ ^+^ and IL-2^+^ were observed in immunodiscordant compared to immunoconcordant and HIV-uninfected controls, including CD4^+^ T-cells with a naïve phenotype, CD4^+^ T_CM_ and CD4^+^ T_EMRA early_ cells (Fig. 4). The same differences were found with all the CMV preparations used. No significant changes were observed in the CD8^+^ T-cell population (data not shown).

Several memory CD4^+^ T-cell subsets produce IFN-γ and IL-2 after p24 stimulation in both immunoconcordant and immunodiscordant individuals, with higher frequency of CD4^+^ HIV-specific T-cells producing IL-2 with a naïve phenotype observed in immunodiscordant individuals compared with immunoconcordant individuals (Fig. 4).

### Association between the magnitude of CMV-specific T-cell responses and T-cell maturation profile

**Figure 5:**
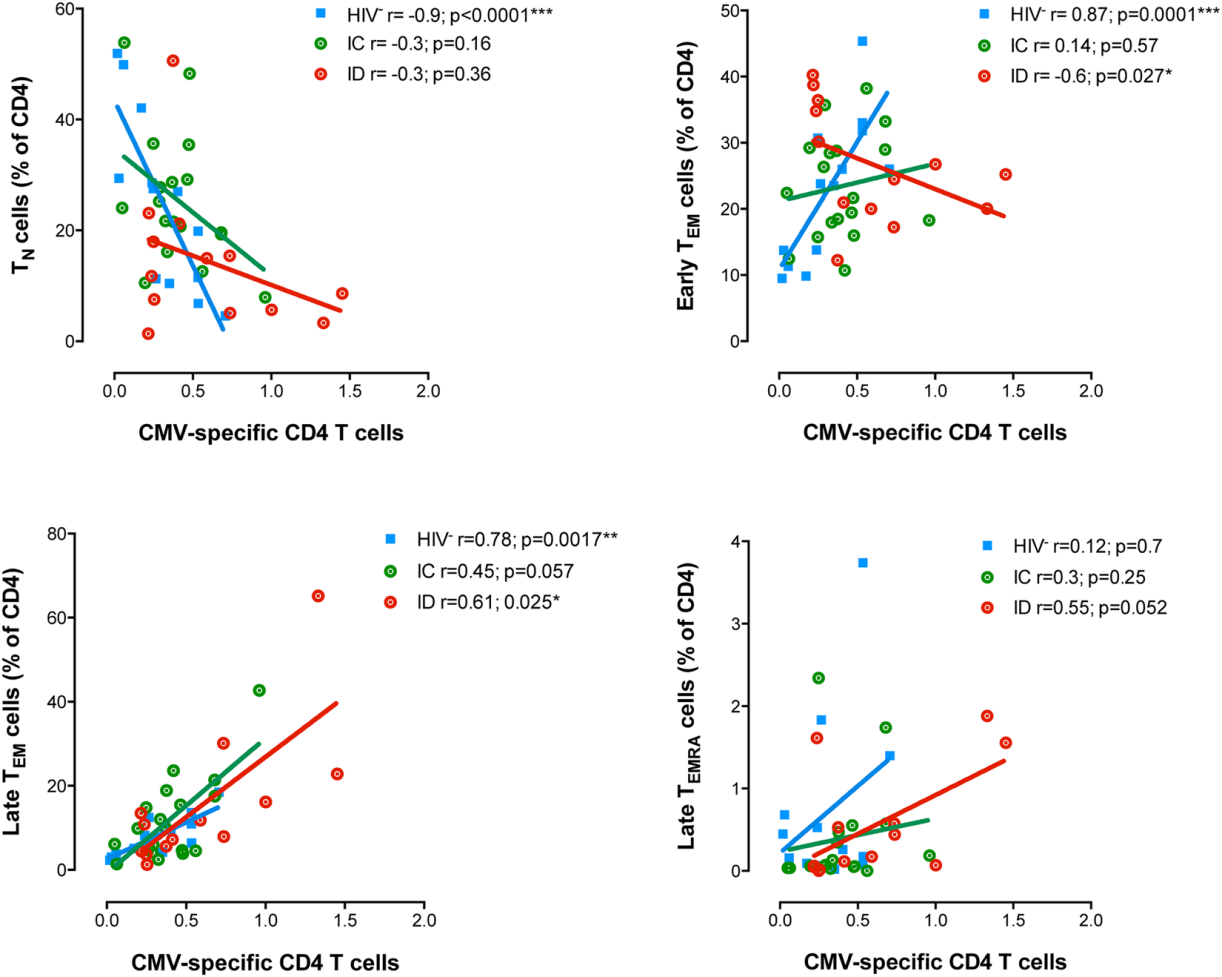
Associations between total CMV-specific CD4^+^ T-cell response and CD4^+^ T-cell subsets. Correlations between total CMV-specific response (Median of total response IFN-γ and IL-2 production of all CMV antigens used) and CD4^+^ T-cell subsets are represented. CMV^+^ HIV-uninfected individuals (blue dots, n = 13), CMV^+^ immunoconcordant individuals (green dots, n = 18) and CMV^+^ immunodiscordant individuals (red dots, n = 13). Linear correlation (Spearman) r and p-values are shown.

As shown in Fig. 1, CMV-seropositivity negatively impacts on the proportion of naïve T-cells and positively impacts on the frequency of effector memory T-cells in HIV-uninfected individuals. Accordingly, in HIV-uninfected individuals we observed a strong negative correlation between the CMV-total response (defined as the mean of the sum of the IFN-γ and IL-2 frequencies from different CMV antigens) and the proportion of CD4^+^ naïve T-cells (Spearman r = -0.9; p < 0.0001) and a positive correlation with the proportions of both CD4^+^ early and late T_EM_ cells (Sperman r = 0.87; p < 0.0001 and Spearman r = 0.78; p = 0.002) (Fig. 5). In contrast, this clear effect seems to be perturbed in HIV^+^ individuals. Indeed, no correlation between the CMV-total response and the proportion of naïve CD4^+^ T-cells in the HIV-suppressed population was found. The correlation with the proportion of CD4^+^ T_EM early_ cells was also lost in immunoconcordant individuals and was reversed in immunodiscordant individuals showing a significant negative value (Sperman r = −0.61; p = 0.03). Analysis of other memory subsets showed a significant positive correlation with the frequency of CD4^+^ T_EM late_ T-cells (Spearman r = 0.61; p = 0.02) and a trend with the proportion of CD4^+^ T_EMRA late_ T-cells (Spearman r = 0.55; p = 0.05) was observed (Fig. 5). No associations were observed between the CMV-response and the composition of the CD8^+^ T-cell compartment in any of the studied population.

### Characterization of CMV and HIV-specific CD4^+^ and CD8^+^ T-cell differentiation profile

**Figure 6:**
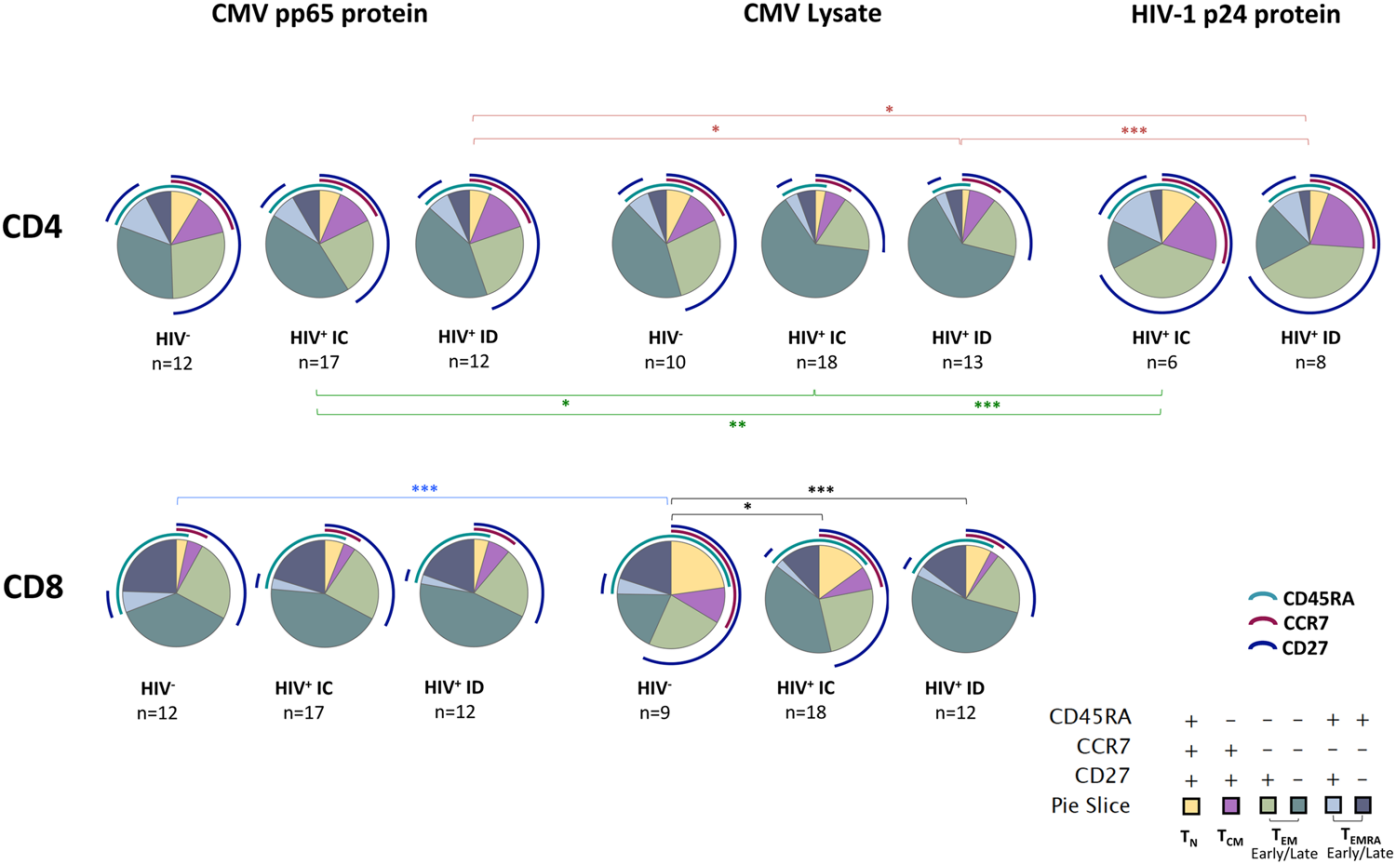
Phenotypic profile of IFN-γ ^+^ virus-specific CD4^+^ and CD8^+^ T-cells. IFN-γ^+^ CMV- and HIV-specific CD4^+^ and CMV-specific CD8^+^ T-cells were determined after stimulation with different CMV antigens and the HIV p24 recombinant protein. The memory maturation profile of the IFN-γ^+^ specific cells was examined for CD45RA, CCR7 and CD27 expression by using a boolean gated strategy. The distribution of CMV- and HIV-specific IFN-γ^+^ response among different subsets was represented using SPICE software. The phenotypic patterns are color-coded and indicated. Significant differences between antigens are depicted in different colors: blue for CMV^+^ HIV^−^ individuals, green for CMV^+^ immunoconcordant (IC) and red for CMV^+^ immunodiscordant (ID). Significant differences between groups (intra-antigen) are also represented (black lines). Statistical testing by permutation performed with SPICE software (*p < 0.05, **p < 0.01, ***p < 0.001).

Finally, an analysis of the differentiation profile of CMV-specific CD4^+^ and CD8^+^ T-cells and HIV-specific CD4^+^ T-cells was performed. No difference between the patterns of response to the pp65 peptide pool and the pp65 protein were observed, therefore only the responses to the pp65 protein, the CMV viral lysate and the HIV-1 p24 recombinant protein are shown (Fig. 6 and Figure S3 for IFN-γ and IL-2, respectively). CMV-specific IFN-γ-producing CD4^+^ T-cells in HIV-negative individuals showed a similar phenotypic pattern of responses with all the stimuli used, with most of the cells displaying a T_EM_ phenotype with an equal response of T_EM early_ and T_EM late_ cells (Fig. 6). Conversely, in HIV-infected individuals a different pattern of response was observed with the different CMV antigenic preparations. The differentiation pattern of the viral lysate-specific CD4^+^ cells among HIV^+^ individuals, in both immunoconcordant and immunodiscordant groups, was significantly skewed towards T_EM late_ cells compared with the differentiation pattern of pp65 protein-specific IFN-γ^+^ cells. Similarly, differences were found in the profile of CMV-specific CD8^+^ IFN-γ ^+^ cells (Fig. 6) and in CD4^+^ and CD8^+^-specific IL-2^+^ T-cells responding to different CMV stimuli and among groups (Figure S3). However, it is important to note that no significant differences were found between immunodiscordant and immunoconcordant individuals with any of the CMV antigens (Fig. 6 and Figure S3). Regarding HIV-specific response, the differentiation profile of both IFN-γ^+^ and IL-2^+^ HIV-specific CD4^+^ T-cells was significantly different to that of CMV, but no differences were observed between immunoconcordant and immunodiscordant individuals. Therefore, immunodiscordant subjects despite displaying a skewed T-cell maturation profile do not present alterations in the differentiation pattern of either CMV- or p24-specific T-cells in comparison with immunoconcordant individuals.

## Discussion

This study provides an analysis of T-cell functionality in a group of HIV-infected individuals with different CD4^+^ T-cell recovery upon virologically suppressive cART. Our data show that even thought immunodiscordant individuals have a skewed T-cell maturation profile^10–13^, T-cell polyfunctionality, assessed by cytokine production after polyclonal and HIV-specific stimulation, is preserved in those individuals. In addition, we have observed that the magnitude of CMV-specific response was differentially associated with state of CD4^+^ T-cell maturation in immunodiscordant and immunoconcordant or HIV-negative groups, likely related to a more frequent or intense subclinical CMV reactivation from latency in immunodiscordant individuals (Gómez-Mora *et al*. PLOS ONE. Accepted Manuscript).

CMV infection, in addition to shape the naïve and memory T-cell repertoire^28–31^, appears to be associated with accelerated T-cell immunosenescence and immune exhaustion, both phenotypes associated with reduced T-cell functionality^18,19^. These CMV-induced phenotypic and functional changes were corroborated in our HIV-uninfected population, with higher level of differentiated CD4^+^ and CD8^+^ T-cells and differences regarding cytokine production in CMV-seropositive individuals compared with CMV-seronegative individuals. Therefore, CMV-seronegative individuals were excluded to avoid the bias in the analysis of functionality of mixed populations (CMV-seropositive and CMV-seronegative individuals) and should be taken into account in future phenotypic and functional characterizations. Indeed, the phenotypic characterization of immunodiscordant individuals, in accordance with our previous reports, showed a decrease in the percentages of both naïve CD4^+^ and CD8^+^ T-cells and an increased frequencies of mature T-cells in comparison with the immunoconcordant group, although the differences were lower than in our previous studies^7,13^ likely due to the exclusion of the CMV-seronegative individuals in the present study. Differences with the HIV-uninfected group were also found, however we cannot exclude that the difference with this group, mainly the decrease in naïve T-cells, could be also associated with age. The HIV-infected population was slightly older than the HIV-uninfected population, although the difference was no statistically significant.

After the non-specific polyclonal stimulation with PMA/ionomycin, a robust production of IFN-γ, IL-2 and TNF-α was displayed in all individuals. Remarkably, CD4^+^ and CD8^+^ T-cell polyfunctionality was not reduced in immunodiscordant individuals compared with immunoconcordant and HIV-negative individuals suggesting that the homeostatic alterations and the increased replicative senescence or exhaustion observed in immunodiscordant individuals per se may not be linked to reduced polyfunctionality. A similar paradox has been reported in healthy older people, who maintain polyfunctionality despite phenotypic alterations, as compared to younger people^32,33^. Quantitatively, immunodiscordant individuals showed a significant enrichment in IFN-γ-producing CD4^+^ and CD8^+^ T-cells and a decrease in monofunctional IL-2-producing T-cells as compared with immunoconcordant and HIV-negative individuals. In line with this data, it has been recently reported that the decrease of mRNA level of IFN-γ after maraviroc administration was associated with the increase in CD4^+^ T-cells in subjects with poor CD4 T-cell recovery^34^. Furthermore, CD8 T cells producing C-C motif chemokine ligand 4 (CCL-4), CD107a and IFN-γ were negatively associated with immune reconstitution^35^. A potential cause could be the disturbed maturational profile displayed by the immunodiscordant individuals; the enrichment in highly-differentiated cells and the low proportions of naïve T-cells as IFN-γ is primarily produced by differentiated memory cells and IL-2 by naïve and central memory T-cells^36^. Potential consequences of the alteration in IFN-γ production could be associated with its polyfunctional effects on immune activation and pro-inflammatory responses by sustaining the chronic immune activation and the apoptosis sensitivity associated with HIV infection (reviewed in ref.^37^). On the other hand, IL-2 is a central regulator of T-cell proliferation, activation and differentiation^38^, thus, slight differences in IL-2 production, also reported by others^12,39^, might be contributing to the poor immune recovery and skewed T-cell maturation observed in immunodiscordant subjects.

CMV-specific T-cell responses are higher in HIV-infected individuals than in HIV-uninfected individuals, particularly those receiving cART^23,40^. In our study, HIV-infected subjects showed a tendency towards higher proportions of CMV-specific IFN-γ and IL-2 secreting CD4^+^ and CD8^+^ T-cells than HIV-uninfected individuals. In addition, we also found changes in the differentiation profile of CMV-specific T-cells between HIV-infected and uninfected individuals stimulated with viral lysate. All these data suggest that HIV-infected individuals could have a different pattern of CMV protein expression, maybe relate to recurrent episodes of subclinical viral reactivation^41^ and which could result in higher CMV-specific responses and a change of CD4^+^ T-cell immunodominance. In the HIV-infected population, the highest CMV-specific T-cell responses were found in immunodiscordant individuals in line with previous work reporting that strong anti-CMV responses are associated with lower CD4^+^ T-cell counts^24^. Moreover, in immunodiscordant individuals the increase in the size of the T-cell response to CMV was associated with an increased proportion of several CD4^+^ T-cell memory subsets responding to CMV having also an impact in the entire memory CD4^+^ T-cell subset distribution. Consistent with a previous report^42^ a correlation between the degree of T-cell differentiation and the size of the CMV-response was observed in HIV-uninfected individuals. However, in contrast with immunoconcordant individuals in whom CMV infection does not seem to have an important role in the CD4^+^ T-cell distribution, in immunodiscordant individuals CMV infection could be involved in the observed skewed T-cell maturation.

The heightened T-cell response against CMV observed in immunodiscordant individuals is not necessarily beneficial. In our study, an association between CMV-specific T-cell response and anti-CMV IgG antibodies was observed with higher level of anti-CMV IgG antibodies in immunodiscordant individuals (Gómez-Mora *et al*., PLOS ONE. Accepted M﻿anuscript) and in line with a recent study, where an association between CMV IgG levels and disease progression has been reported^27^. It has been demonstrated that circulating anti-CMV IgG antibodies and CMV-specific T-cell responses are associated with cardiovascular diseases and physical function impairment^23,25,26,43^, suggesting that in HIV^+^ individuals with poor CD4^+^ T-cell recovery the high CMV-specific response, probably due to episodes of CMV reactivation mainly in the genital tract^41,44^, may have an impact in the higher mortality and morbidity observed in those individuals.

As expected, when compared to CMV, low HIV-1-specific CD4^+^ T-cells were detected in most ART-suppressed individuals^45^, with a distinct differentiation profile^46–48^. It has been previously shown that individuals with poor CD4^+^ T-cell recovery have reduced Gag-specific IFN-γ ELISPOT response^11^ and poor CD8^+^ lymphoproliferative responses to Gag^39^. However, a higher IL-2-secretion after gp120-stimulation by CD4 T-cells in individuals with a low-level CD4 T-cell repopulation has been also reported^49^. Using a p24 protein, we did not find any difference in the total IFN-γ^+^ or IL-2^+^ HIV-specific immune response between immunoconcordant and immunodiscordant individuals. Surprisingly we found increased frequencies of IL-2^+^ HIV-specific CD4^+^ T-cells with a naïve phenotype in immunodiscordant individuals. It has been reported that higher frequencies of HIV Gag-specific IFN-γ^+^IL-2^+^ CD4^+^ T-cells are strongly associated with higher cell-associated HIV DNA levels in HIV controllers^50^ and CMV reactivation has been associated with higher levels of HIV DNA in blood cells^44^. In cART-treated HIV-positive individuals a trend for association between HIV-specific CD4^+^ T-cells expressing IL-2 and the total HIV DNA in resting CD4^+^ T-cells has also been described^51^. Therefore, the higher levels of proviral DNA that have been found in immunodiscordant individuals^52^ could be associated with a higher percentage of IL-2^+^ HIV-specific CD4^+^ T-cells, which are more likely to be infected with HIV^53^ and related to CMV infection as previously suggested^44^. The use of a protein instead of a pool of peptides only allowed us to evaluate the HIV-specific CD4^+^ T-cell responses, so we can not rule out that there are also differences in the IFN-γ production by CD8^+^ T-cells between immunodiscordant and immunoconcordant individuals.

One limitation of this study is the low number of individuals analyzed, especially for the study of HIV-specific responses. In addition, polyfunctionality (measured by the expression of IFN-γ, IL-2 and TNF-α) was only determined following the non-specific stimulation. A polyfunctional profile, including cytolytic markers as perforin and granzyme B and the degranulation marker (CD107), have not been analyzed and it has been demonstrated that the highly polyfunctional antigen-specific cells are the most potent effectors and that could be correlated with clinical outcome^54–56^.

Taken together, our results indicate that HIV-infected ART-suppressed individuals with poor immune recovery displayed a maintained global T-cell polyfunctionality despite having severe homeostatic alterations and increased levels of replicative senescence and exhaustion. Furthermore, in immunodiscordant individuals, asymptomatic CMV infection, that has been previously described as a major contributor to the increased immune activation and senescence resulted in a heightened CMV-specific immune response that may be contributing to the skewed T-cell maturation and the higher risk of clinical progression observed in those individuals. Future studies are needed to determine if persistent CMV replication could be targeted as a strategy to reduce the size of specific immune response and reduce the CMV-associated immune pathology.

## Methods

### Subjects

A total of 43 HIV-infected individuals were recruited for the study. Inclusion criteria for all participants were confirmed diagnosis of HIV infection, continuous cART with sustained undetectable HIV-1 RNA (plasma viral load < 50 copies/ml) for at least the past 2 years (minimum of four determinations during this time period) and good antiretroviral treatment (ART) adherence. Individuals were classified as immunoconcordant (favorable virologic and immunologic response) when CD4^+^ T-cell counts were above 400 cells/µL and as immunodiscordant individuals (favorable virologic response but unsatisfactory immunologic response) if CD4^+^ T-cell counts were persistently less than 350 cells/µL. For comparative purposes a control group of 21 HIV-uninfected individuals was also analyzed. The institutional review board of the Hospital Germans Trias i Pujol approved the study (EO code: EO-07-024). The methods were carried out in accordance with the Declaration of Helsinki. Written informed consent was obtained from all participants.

### Determination of anti-CMV IgG levels

The CMV-specific IgG antibodies were measured using the semi-quantitative chemiluminiscent immunoassay (Synlab Diagnostics, Barcelona, Spain). Samples determined as <12U/ml were considered negative, determinations between 12 and 14U/ml were undetermined, and >14U/ml were considered positive samples.

### CMV viral load detection by RT-PCR

CMV viral load was detected by extraction of DNA from urine samples (QIAmp Viral DNA Kit, Qiagen) and analyzed by real time PCR (Realquality RQ-CMV AB Analitica) according to the manufacturer’s instructions (AB Analitica). The assay detected CMV DNA in a linear range from 600 copies/mL to 6 × 10^8^ copies/mL.

### Analysis of polyfunctionality of T-cell responses

*Ex vivo* expression of IFN-γ, IL-2 and TNF-α by CD4^+^ and CD8^+^ T-cells was assessed by multicolor flow cytometry analysis. In brief, freshly isolated PBMCs (2 × 10^6^ cells per condition) were stimulated in polypropylene tubes with PMA (6.25ng/mL) plus ionomicyn (0.6 μM) and with a recombinant HIV p24 capsid protein (5.5 µg/ml, Protein Sciences Corp) to evaluate global T-cell functionality and HIV-specific response, respectively. In addition, to characterize more accurately the CD4^+^ T-cell functionality, the most impaired population in immunodiscordant individuals, different CMV antigenic preparations optimal for stimulating a robust response especially from CD4^+^ T-cells were included^57–59^: a purified CMV viral lysate (0.5 µg/ml, ZeptoMetrix, Buffalo, NY), a pool of overlapping peptides (15-mer) covering the whole HCMV pp65 protein (30 µg/ml, obtained through the NIH AIDS Reagent Program, Division of AIDS, NIAID, NIH^59–61^ and a recombinant CMV-pp65 protein (7.5 μL per sample, Milteny Biotec, Madrid, Spain). An unstimulated sample control was also included. Co-stimulatory anti-CD49d and anti-CD28 (1 µg/ml, Becton Dickinson) were added to all tubes and after 2 hours of incubation at 37 °C, Brefeldin A and Monensin were added according to the instructions of the manufacturer (both from Becton Dickinson) and cells were incubated for an additional 6 h. Cells were then washed and labeled with the Fixable Yellow Dead Cell Stain Kit (Invitrogen) to identify dead cells, washed and stained with the following extracellular antibody combination: V450-CD3, V500-CD8, Alexa700-CD45RA, PE-Cy7-CCR7 and BV605-CD27 (all from Becton Dickinson) and eFluor650-CD4 (e-Biosciences). Subsequently, cells were fixed and permeabilized with Cytofix/Cytoperm (Becton Dickinson) and intracellular staining was carried out with FITC-IFN-γ, APC-IL-2 and PE-TNF-α (all from Becton Dickinson). After intracellular staining, cells were washed and acquired in LSR-Fortessa flow cytometer (Becton Dickinson) in the Cytometry Core Facility at Germans Trias i Pujol Research Institute.

### Analysis of flow cytometry data

Flow cytometry data were analysed using FlowJo software (9.8 v; TreeStar, Portland, OR, USA). Initial gating was performed on the forward scatter height vs. forward scatter area to remove doublets, and then living T lymphocytes were gated according morphological parameters and cell viability followed by identification of CD4^+^ and CD8^+^ T-cells (Figure S1). Mitogen PMA/ionomycin stimulation leads to a rapid downregulation of membrane expression of CD4^+^^62,63^, therefore CD3^+^ CD8^-^ T-cells were used to analyzed the CD4^+^ T-cell response in the non-specific analysis. Finally, CD4^+^ and CD8^+^ T-cell subpopulations were identified by the expression of CD45RA, CCR7 and CD27 (Figure S1). For the cytokine production analysis, individual gates (set on the basis of the unstimulated control) were made to identify positive responses (Figure S1). FlowJo Boolean gating was performed to create a full array of possible combinations of up to 8 response patterns from the CD4^+^ and CD8^+^ T-cell gate. After subtracting the background response (with co-stimulation but no antigen), a positive CD4^+^ and CD8^+^ T-cell cytokine response was defined as at least 40 positive events (to minimize the possibility of error due to a low number of events when further subdividing these cells based on their phenotypic profile). Polyfunctionality was represented visually using Pestle (v1.7) and SPICE (v5.35) software (provided by the National Institutes of Health, Mario Roederer, ImmunoTechnology Section, Vaccine Research Centre, National Institutes of Health, Bethesda, MD, USA)^64^. In addition to SPICE analysis, we used the polyfunctional index (PI) defined by Larsen *et al*.^65^ (Funky Cells Toolbox ^66^), which numerically evaluates the degree and variation of polyfunctionality and allows comparative statistical tests.

Differentiation profile of IFN^+^ and IL-2^+^ CD4^+^ and CD8^+^ T-cells were created by FlowJo boolean gating and subsequent SPICE analysis. In addition, functionality of each of the CD45/CCR7/CD27 differentiation subsets was assessed by the determination of IFN-γ^+^ and IL-2^+^ cells within those subsets. In the CMV- and HIV-specific response we do not report on TNF-α production to avoid false-positive results because a low signal-to-background ratio was found in the staining.

### Statistical analysis

Statistical analysis and graphical presentation were performed using Prism software (5.0av; GraphPad). To compare pie charts, we used the SPICE permutation (10,000 permutations) analysis. Mann-Whitney U nonparametric test was used for comparing data among specific responses (*p < 0.05, **p < 0.01, ***p < 0.001). Spearman’s correlation coefficient was calculated to identify associations between variables.

## Electronic supplementary material

Supplementary Information
