# Supplementary material for: Preserved immune functionality and high CMV-specific T-cell responses in HIV-infected individuals with poor CD4+ T-cell immune recovery
Source: Sci Rep. 2017 Sep 15;7:11711. doi: 10.1038/s41598-017-12013-2 (PMC5601464; doi:10.1038/s41598-017-12013-2)
Supplement: Supplementary file 1 — Supplementary Information [file 41598_2017_12013_MOESM1_ESM.pdf]

Preserved immune functionality and high CMV-specific T-cell responses in HIV-infected individuals with poor $CD4^+$ T-cell immune recovery

Elisabet Gómez-Mora[1], Elisabet García[1], Victor Urrea[1], Marta Massanella[1,2], Jordi Puig[3], Eugenia Negredo[3], Bonaventura Clotet[1,3,4], Julià Blanco[1,4] and Cecilia Cabrera[1]

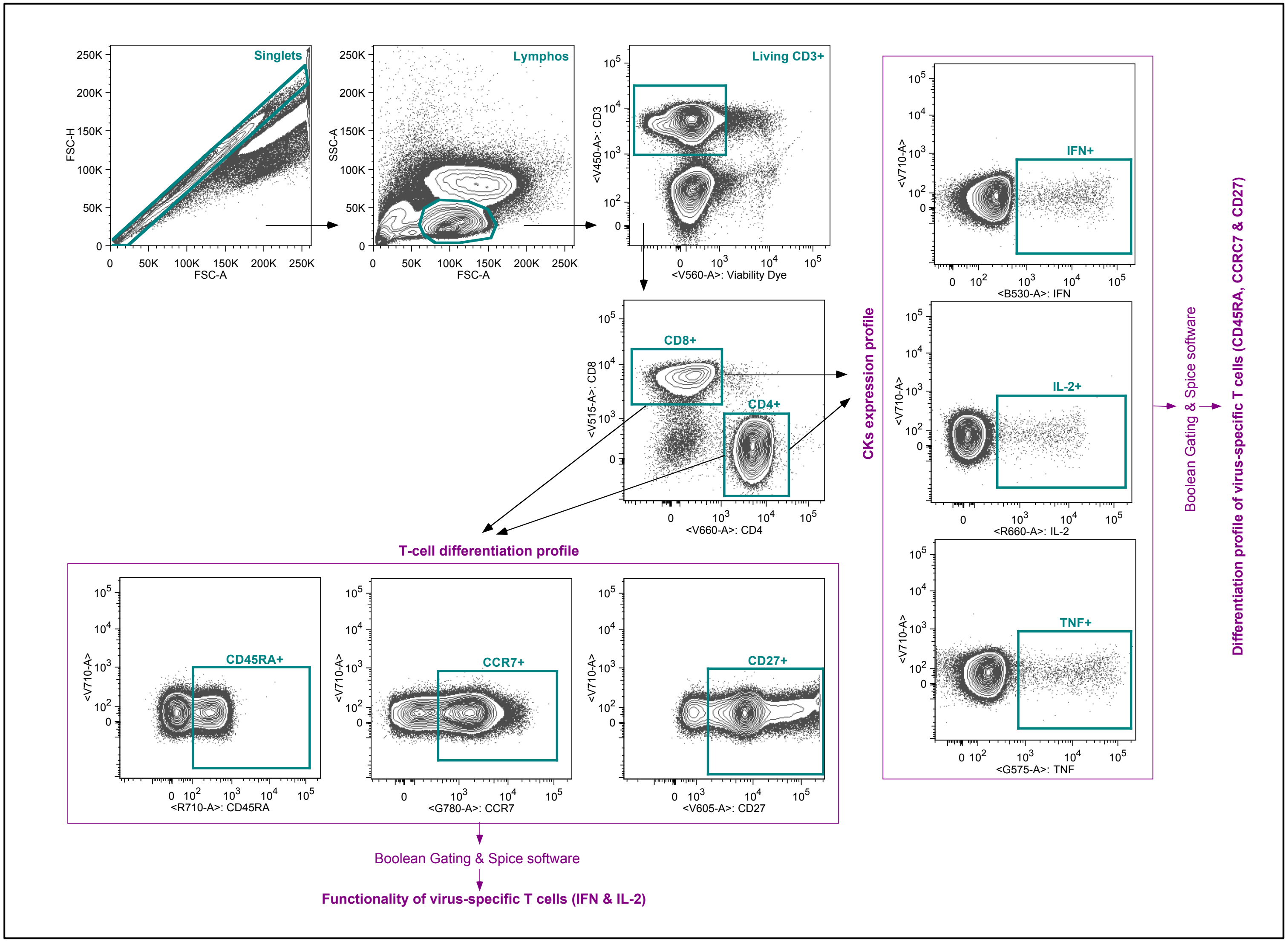

Figure S1. Gating strategy for immune phenotyping and cytokines expression. First, living T lymphocytes were gated according morphological parameters and cell viability. Combinations of CD45RA, CCR7, and CD27 expression were created by boolean gating to determine the distinct $CD4^+$ and $CD8^+$ T-cell subsets (1). Functionality of different $CD4^+$ and $CD8^+$ T-cells subsets was analyzed determining the proportion of IFN-γ^+ and IL-2^+ CMV- and HIV- specific within distinct $CD4^+$ and $CD8^+$ T-cell subsets (2). Expression of IFN-γ, IL-2 and TNF-α and their combinations were also analyzed by boolean gating to determine the specific response within $CD4^+$ and $CD8^+$ T-cells (3). The distribution profile of IFN-γ^+ and IL-2^+ CMV- and HIV- specific $CD4^+$ and $CD8^+$ T-cells was measured using Boolean gating (4). Analysis was carried out using FlowJo and SPICE software (9.2.3v).

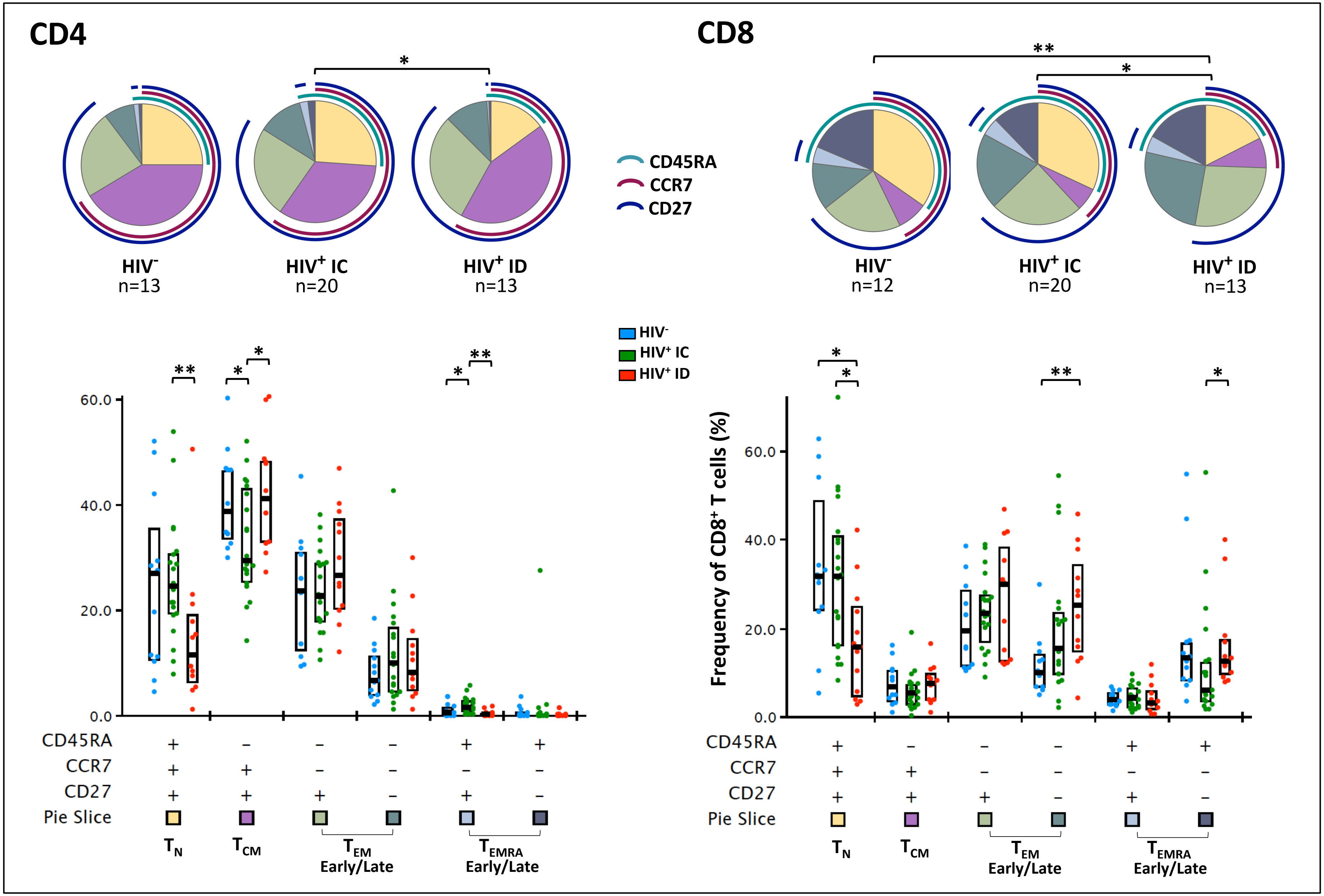

Figure S2. T-cell differentiation profile in HIV-negative, immunoconcordant and immunodiscordant individuals. The differential expression of CD45RA, CCR7 and CD27 by $CD4^+$ and $CD8^+$ T-cells was analysed by boolean gating. Pie charts illustrate relative proportions of each of the combinations of markers in HIV-negative, HIV^+ immunoconcordant (IC) and HIV^+ immunodiscordant (ID) individuals (statistical testing by permutation was performed with SPICE software). Each phenotype (defined by a specific combination of markers) is shown beneath the X-axis (T_N: naïve cells, T_{CM}: central memory, $T_{EM\ early/late}$: effector memory and $T_{EMRA\ early/late}$: effector memory RA cells. Median and IQR are represented. Differences were tested using Mann-Whitney test (*p<0.05, **p<0.01).

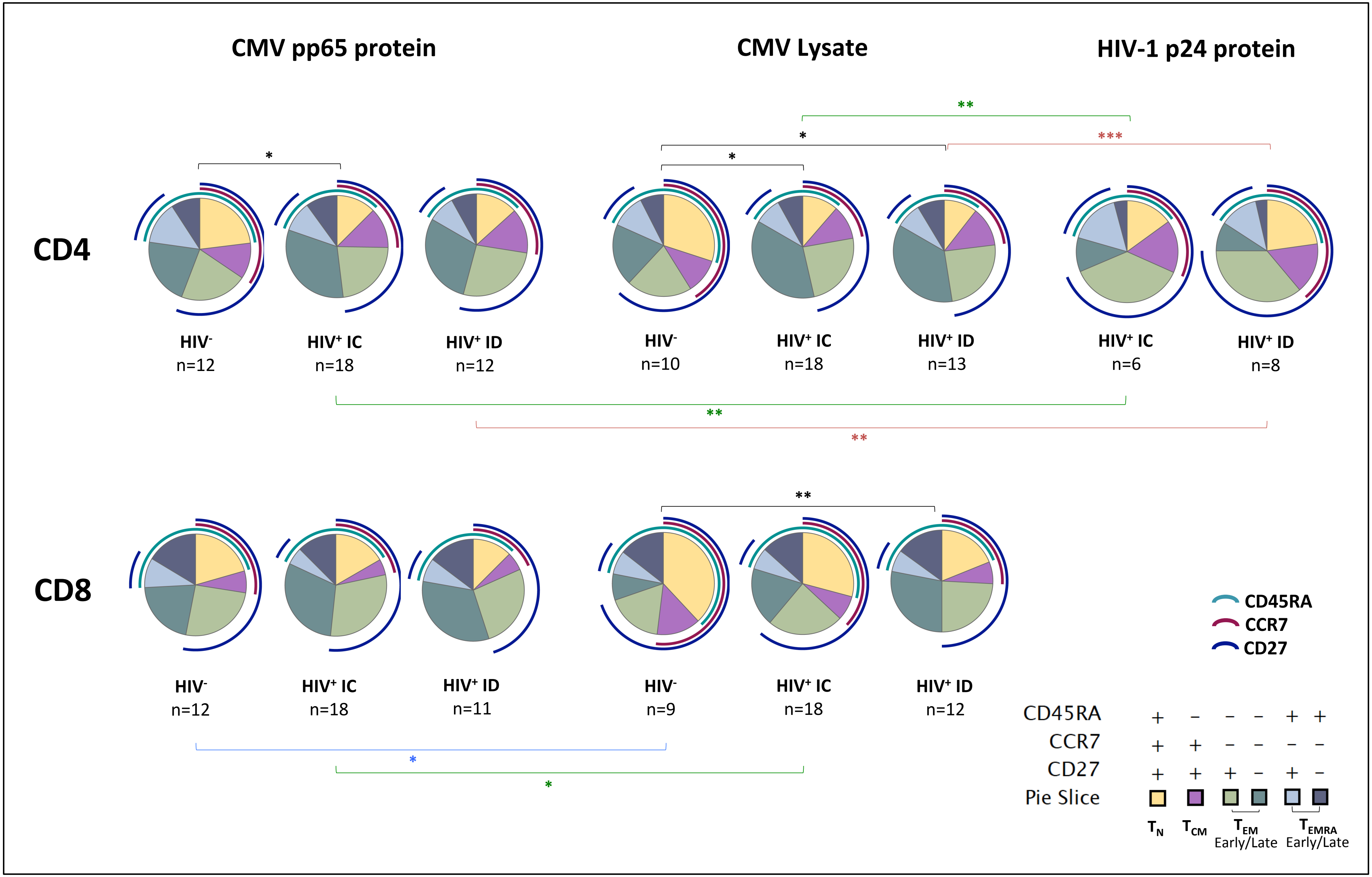

Figure S3. Differentiation profile of IL-2$^+$ virus-specific CD4$^+$ and CD8$^+$ T-cells. IL2$^+$ CMV and HIV-specific CD4$^+$ and CMV-specific CD8$^+$ T-cells were determined after stimulation with all CMV-derided stimuli and the HIV-p24 protein. The phenotypic profile of the responding T-cells was assessed by boolean gating. The phenotypic patterns are color-coded and indicated. Significant differences between antigens are depicted in different colors: blue for HIV$^-$ individuals, green for immunoconcordant (IC) and red for immunodiscordant ID). Significant differences between groups (intra-antigen) are also represented (black lines). Statistical testing by permutation was performed with SPICE software.
